# Minimally invasive surgery for placement of a subcutaneous EEG implant

**DOI:** 10.3389/fsurg.2023.1304343

**Published:** 2023-11-09

**Authors:** Bjarki D. Djurhuus, Pedro F. Viana, Esben Ahrens, Sofie S. Nielsen, Harishchandra L. Srinivasan, Mark P. Richardson, Preben Homøe, Harutomo Hasegawa, Ali A. Zarei, Pia L. K. Gauger, Jonas Duun-Henriksen

**Affiliations:** ^1^ Department of Otorhinolaryngology and Maxillofacial Surgery, Zealand University Hospital, University of Copenhagen, Koge, Denmark; ^2^ Department of Clinical Medicine, University of Copenhagen, Copenhagen, Denmark; ^3^ School of Neuroscience, Institute of Psychiatry, Psychology and Neuroscience, King’s College London, London, United Kingdom; ^4^ Faculty of Medicine, University of Lisbon, Lisbon, Portugal; ^5^ T&W Engineering A/S, Lillerød, Denmark; ^6^ UNEEG medical, Alleroed, Denmark; ^7^ Neurosurgery Department, King’s College Hospital NHS Foundation Trust, London, United Kingdom

**Keywords:** subcutaneous EEG, minimally-invasive surgery, ultra long-term monitoring, epilepsy, sub-scalp

## Abstract

**Background:**

A new class of subcutaneous electroencephalography has enabled ultra long-term monitoring of people with epilepsy. The objective of this paper is to describe surgeons' experiences in an early series of implantations as well as discomfort or complications experienced by the participants.

**Methods:**

We included 38 implantation procedures from two trials on people with epilepsy and healthy adults. Questionnaires to assess surgeons' and participants’ experience were analyzed as well as all recorded adverse events occurring up to 21 days post-surgery.

**Results:**

With training, the implantation could be performed in approximately 15 min. Overall, the implantation procedure was considered easy to perform with only 2 episodes where the implant got fixated in the introducing needle and a new implant had to be used. The explantation procedure was considered effortless. In 2 cases the silicone sheath covering the lead was damaged during the explantation, but it was possible to remove the entire implant without leaving any foreign body under the skin. Especially in the trial on healthy participants, a proportion experienced adverse events in the form of headache or implant-pain up to 21 days post-operatively. In 6 cases, adverse events contributed to the decision to explant and discontinue the study: Four of these cases involved implant pain or headache; One case involved a post-operative local infection; and in one case superficial lead placement resulted in skin perforation a few weeks after implantation.

**Conclusion:**

The implantation and explantation procedures are considered swift and easy to perform by both neurosurgeons and ENT surgeons. The implant is well tolerated by most participants. However, headache or pain around the implant can occur for up to 21 days post-operatively as anticipated with any such surgery. The expected benefits from the implant should always outweigh the potential disadvantages.

## 1. Introduction

While continuous long-term monitoring of the heart has been widely used for many decades, continuous monitoring of the brain was until recently only possible for up to a few weeks (1, 2). In 2019, the first minimally-invasive brain-monitor was CE-marked for ultra long-term recording of two-channel electroencephalography (EEG), the 24/7 EEG™ SubQ (UNEEG medical, Alleroed, Denmark), and other developments are following (1, 2). The novelty lies in the fact that patients are able to record EEG in their everyday life with a discreet and unobtrusive device, thereby enabling objective insights to the brain in areas such as epilepsy (3), sleep disorders (4), diabetes (5), brain-computer interfaces (6) or basic neuroscience research (7). Other continuous EEG devices are all with intracranial leads, and while they provide excellent signal quality, they are also very invasive (8–10).

It is well known, and expected, that signal hampering artifacts are more common in the home environment compared to the shielded hospital setting (11). People are less physically active in an isolated hospital recording compared to everyday life, which creates artifacts in the EEG. Furthermore, scalp electrodes might dislocate or dry out if not serviced daily which creates more artefacts. Conversely, investigations of subcutaneous EEG show a high signal quality in real life recordings (12, 13) with clear identification of physiological morphologies such as electrographic seizures, interictal epileptiform discharges, sleep spindles, K-complexes, and deep sleep slow waves (4, 14).

The objective of this paper is to describe the surgeons' and users' experiences with an early series of implantations.

## 2. Materials and methods

### 2.1. Study population

Included in this study were all participants from the “Sleep in the Ultra Long-Term Prospective study” (ULTS) and the “Subcutaneous EEG: Forecasting of Epileptic Seizures study” (SUBER).

**The ULTS study** was set up to study the ultra-long-term sleep variability in a cohort of healthy adults as well as for development of an automatic sleep stage algorithm (ClinicalTrials.gov Identifier: NCT04513743, Research ethical committee reference: SJ-778). A total of 25 healthy volunteers were recruited for implantation with the subcutaneous EEG system and agreed to record their EEG every night for one year. Twenty volunteers completed the trial. The volunteers were paid for their inconvenience in accordance with ethical committee guidelines. All implantations and explantations were performed at the Department of Otorhinolaryngology and Maxillofacial Surgery, Zealand University Hospital, Koege (ZUH), Denmark by two different ear-nose-throat (ENT) surgeons in the period September 2020 to June 2022. The study was conducted according to ISO14155:2011—Good Clinical Practice for medical devices.

**The SUBER study** was set up to study the feasibility of forecasting epileptic seizures based on two-channel, ultra long-term, subcutaneous EEG (ClinicalTrials.gov Identifier: NCT04061707, Research ethical committee reference: 19/LO/0354). A total of 12 people with drug-resistant epilepsy and at least 20 seizures per year according to own diary were implanted with the subcutaneous device. Two patients dropped out before recording usable data (at least 21 days of data). One patient agreed to be reimplanted during the study providing a total of 13 implantation procedures for this investigation. All surgical procedures were performed at the Department of Neurosurgery, King's College Hospital, London, United Kingdom (KCH). The procedures were performed by three different members of the neurosurgical team in the period July 2019 to March 2023.

### 2.2. The device

**Figure 1:**
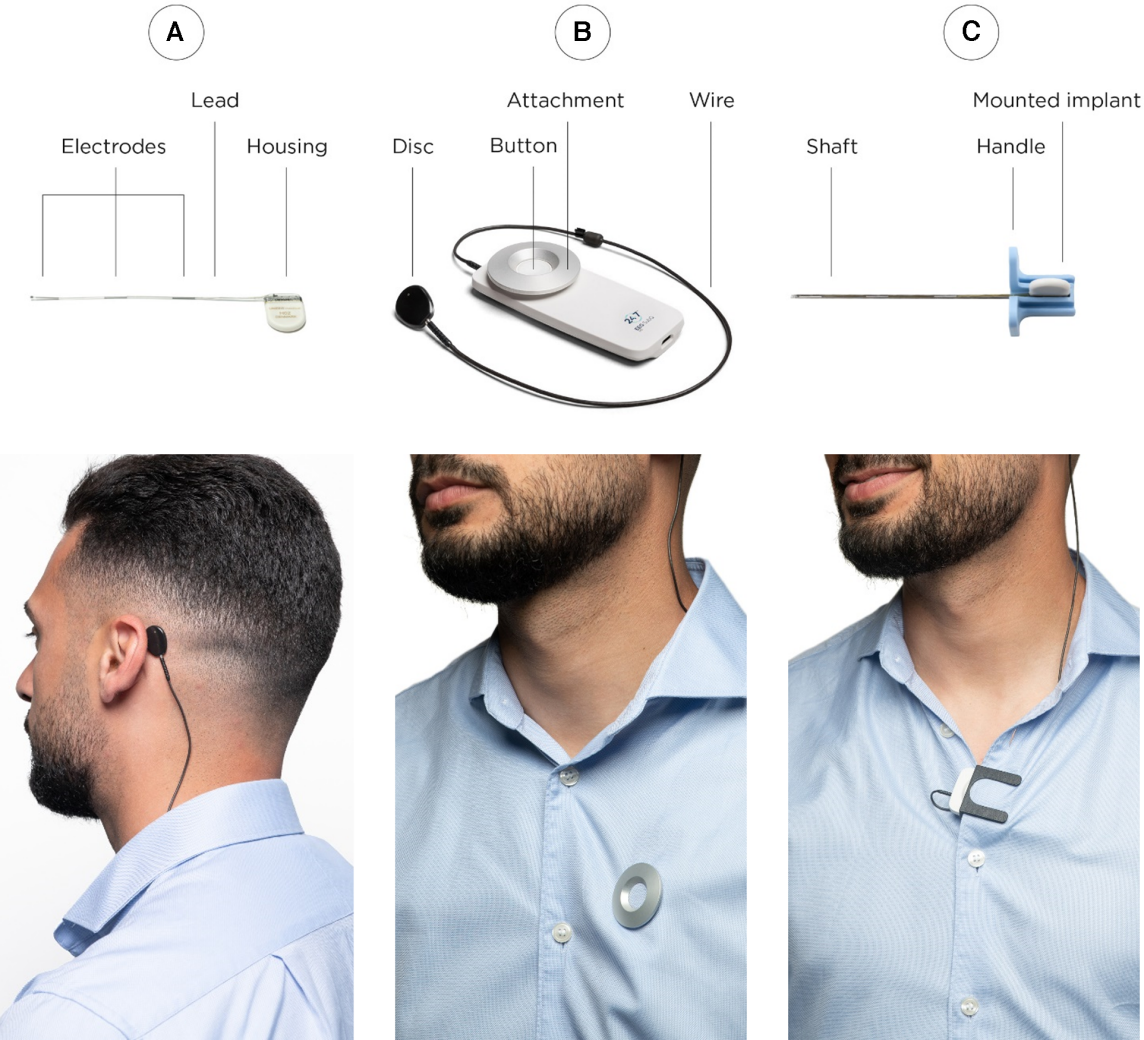
The subcutaneous EEG system consists of two physical parts; the implant (**A**) and the recorder (**B**). The implantation procedure is performed with the use of a dedicated introducing needle (**C**). In the lower row, it is illustrated how the disc of the external recorder is attached with an adhesive pad (left) in close transcutaneous alignment with the housing of the implant. The housing of the recorder can be attached with either a magnet (middle) or a clip (right) if the patient has other active implantable devices sensitive to magnets.

The investigated subcutaneous EEG system comprises of two components: The implant (Figure 1A) and the external recorder (Figure 1B). In addition, an introducing needle (Figure 1C) is used to aid the implantation procedure. The implant consists of a housing (24 × 17 × 3.3 mm) made of titanium, tungsten, gold, and ruby with a ceramic casing and a lead (103 × 1.1 mm) made of silicone with a stranded core and 3 platinum-iridium electrodes (2 active and 1 reference). Each electrode is 10 mm long and they have a center-to-center distance of 35 mm. For ease of implantation the implant is positioned in a dedicated open introducing needle (125 × 24 mm, of which 97 mm is the shaft length, outer diameter 2.11 mm, inner diameter 1.55 mm). The implantation procedure is described in detail in the following section.

The external recorder consists of a house (89.9 × 37.5 × 10.9 mm), a 37 cm wire and a disc for wireless inductive powering up the implant and receival of data. The disc is attached to the skin with an adhesive pad (Figure 1 left) and requires close transcutaneous alignment with the housing of the implant for connection. The external recorder exists in two variants: One with an attachment magnet and one with an attachment clip in case the participant has other active implantable devices sensitive to magnets (Figure 1 middle and right).

The subcutaneous EEG system is indicated for measuring and recording of electrical activity of the brain (EEG) through electrodes implanted subcutaneously in the tissue between the skull and the skin. It is intended for subjects where single location, continuous, ultra long-term (more than two weeks) EEG recordings are indicated to aid in monitoring and diagnosis of diseases or conditions that alter the EEG. The intended users of the product are individuals aged 18 and above. Contraindications according to the manufactures instruction for use are (15):

– Subjects with cochlear implant(s).
– Subjects involved in therapies with medical devices that deliver electrical energy in the area around the implant.
– Subjects at high risk of surgical complications, such as active systemic infection and hemorrhagic disease.
– Subjects who are unable (i.e., mentally or physically impaired) or do not have the necessary assistance to properly operate the device system.
– Subjects who have an infection at the site of device implantation.
– Subjects who operate MRI scanners.
– Subjects with a profession/hobby that includes activity imposing extreme pressure variations (e.g., diving or parachute jumping). NB: diving/snorkelling is allowed to 5 meters depth.

### 2.3. The implantation procedure

**Figure 2:**
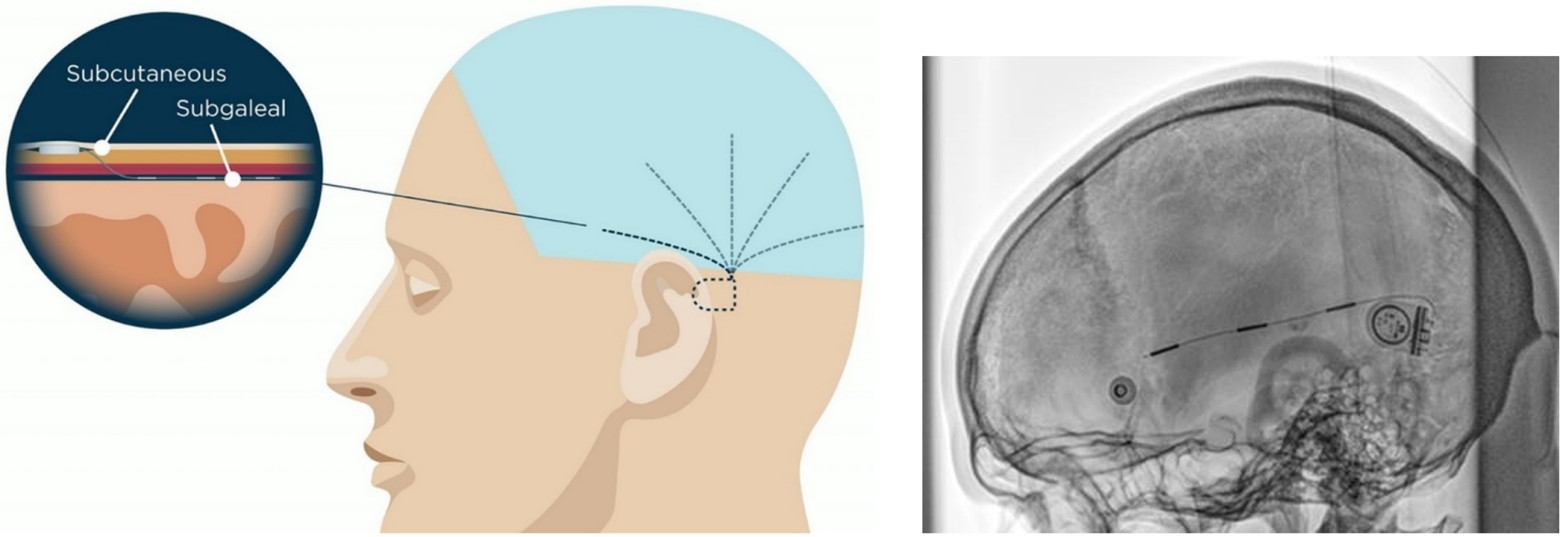
Left: the lead of the implant is placed deep to the galea while the housing is placed subcutaneously. It is possible to direct the implant lead so that it covers various part of the brain. In the figure, five different placements are suggested for illustrative purpose: temporal, fronto-temporal, central, parietal or occipital. Note that the device only has one lead. Right: post-implantation x-Ray of a placement over the superior part of the temporal lobe. The housing is bend approximately 90° to pull the lead more posteriorly. It can be bend up to 180°. x-ray in courtesy of Prof. Coenen and Prof. Schulze-Bonhage, University Hospital Freiburg, Germany.

The implant housing is recommended to be placed under the skin behind the ear no matter the position of the lead (see Figure 2). Here, the housing rests on a relatively flat and stable part of the cranium close to, but preferably outside, the hairline. This position eases the device management and may make the need of regular shaving unnecessary. The lead with the three electrodes is also placed under the skin and can be directed to cover a unilateral region of the cranial convexity over the temporal, frontal, parietal or occipital regions. For placement of the housing of the implant, it is important to consider whether the participant is using glasses and/or hearing aids. In the SUBER study, conducted on people with epilepsy, previous scalp EEG recordings were scrutinized to find the optimal location of the lead. Ideally, the lead should be placed over the area of maximal signal-to-noise ratio during a seizure, and not necessarily over the precise seizure onset zone. While inspecting the scalp EEG recordings, new channel derivations were constructed if necessary to identify this region. Also, if necessary, the neurologist confirmed the lead placement in the operating room with the neurosurgeon. To direct the position of the lead more proximally to the housing, the housing can be rotated to bend the lead as shown on the x-ray in Figure 2. X-rays were not part of the standard procedure. Dependent on the hair growth of the participant, some may have to shave every 3rd to 4th week to be able to attach the adhesive pad on the external disc.

If bilateral recordings are needed, two devices can be implanted, one on each side of the head; this also means the participant will need to wear two external devices.

**Figure 3:**
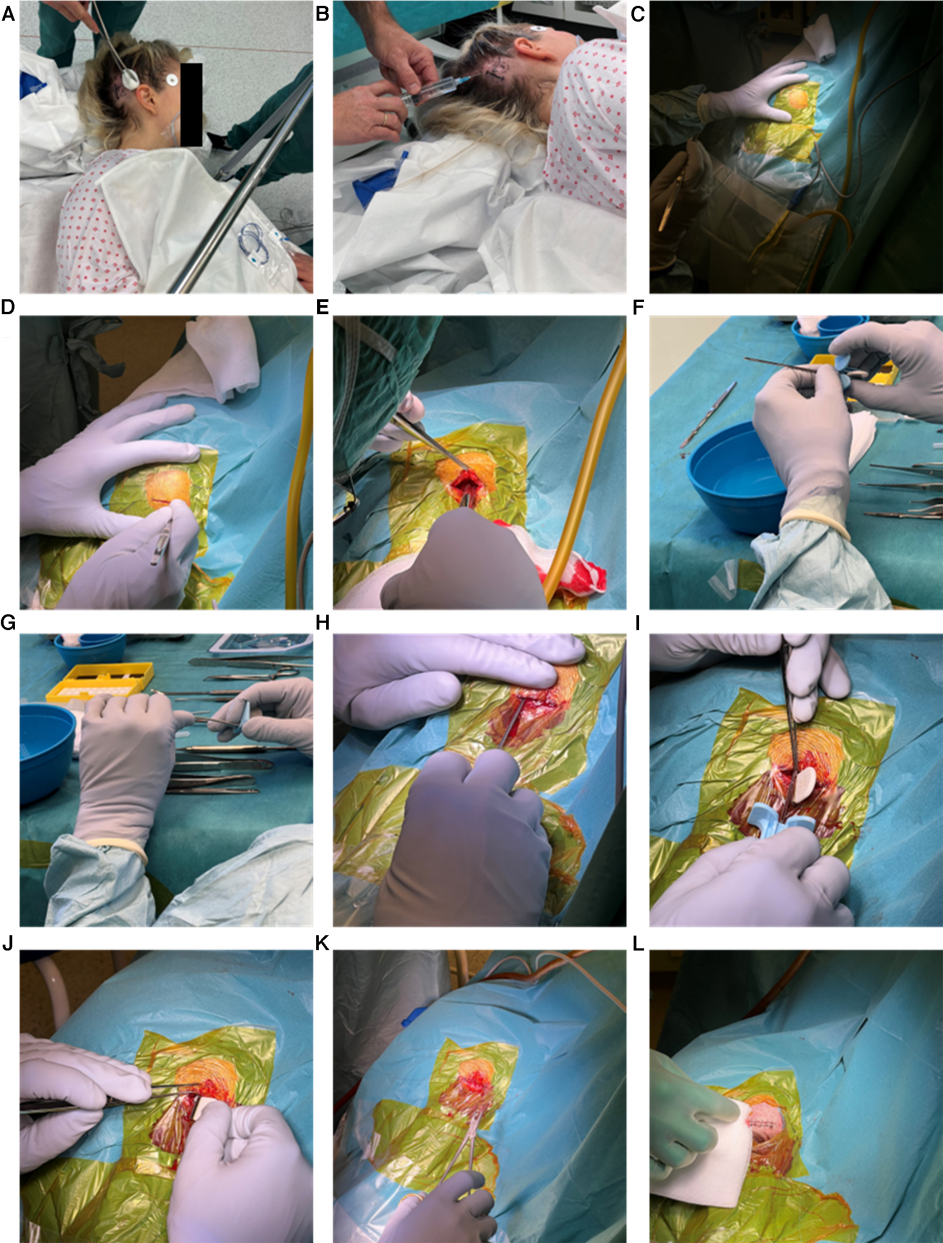
The implantation procedure as described in the main text (Section 2.3).

The surgical guide provided by the manufacturer was followed (15). The following description is a typical temporal implantation performed in the ULTS study:

1) The participant was positioned supine with the head turned to the contralateral side of where the implant should be positioned (Figure 3A). A small patch of hair was removed, and the implant position was sketched on the skin (Figure 3A).
2) Local anesthesia (5 ml lidocaine with adrenalin. Notice that surgical guide does not specify type of anesthesia nor dose, this is mentioned because we describe the procedure followed in the ULTS study) was applied corresponding to the desired location of the housing, the incision line and along the planned trajectory of the lead (Figure 3B).
3) Skin preparation and draping was performed (Figure 3C). A drape sheet that allows inspection and palpation in the desired area of the lead trajectory during insertion may be preferred.
4) A linear approximately 25 mm vertical incision was made behind the hairline and at least 10 mm behind the planned posterior border of the housing (Figure 3D).
5) A subcutaneous pocket for the housing was created (Figure 3E).
6) The implant was fitted in the introducing needle (Figure 3F).
7) The introducing needle was bent carefully to fit the curvature of the participant's skull (Figure 3G).
8) Using the introducing needle, the epicranial aponeurosis was penetrated and the lead was inserted in the subgaleal space. The introducing needle was pushed until the tip in the vertical plan was approximately 6 cm in front of the posterior border of pinna's attachment in a safe distance from the temporal branches of the facial nerve (Figure 3H).
9) The introducing needle was withdrawn carefully leaving the lead *in situ* with the help of a blunt tweezer (Figure 3I).
10) The housing was inserted into the retroauricular subcutaneous pocket (Figure 3J). The housing should be at least 5 mm behind the pinna and use of glasses should be kept in mind.
11) The skin was closed with 4–0 surgical suture (Figures 3K,L). A pressure bandage was applied and kept for the rest of the day to prevent seroma/hematoma.
12) Sutures were removed after approximately 10 days.

### 2.4. The explantation procedure

The explantation procedure resembled the implantation. Local anesthesia was applied around the housing and the cicatrix from the implantation procedure. The prior incision was reused. Care was taken not to damage the implant, especially the silicone sheathing of the lead. The implant was removed by gently pulling the implant housing on the white ceramic part with a Murphy Pean Forceps making sure the lead followed without resistance. After removal, the implant was inspected, and care was taken that no remnants were left. Closure and bandaging were similar to the implantation procedure.

### 2.5. Data collection

In both the ULTS and the SUBER study three questionnaires related to the implantation procedure were filled out:

1) A 7-item questionnaire filled out by the surgeon immediately after the implantation procedure.
2) A questionnaire filled out by the participant asking about discomfort related to the implant ≥2 months after implantation and again at study end before explantation.
3) A 6-item questionnaire filled out by the surgeon immediately after the explantation procedure.

In addition, adverse events and device deficiencies were registered continuously by project staff upon appearance. Data on adverse events assessed related to the surgical procedure were obtained in several ways: E.g., if the participant mentioned headache during a phone call, a planned control visit or on removal of sutures. The same participant could therefore contribute with the same adverse on several occasions. Only adverse events recorded within the first 21 postoperative days were assessed relevant to the surgical procedure. All adverse events were coded according to the International Medical Device Regulators Forum (IMDRF) for Adverse Events Terminology (release number 2022).

### 2.6. Data analysis

Full analysis set, defined as all data obtained from all participants enrolled in the study was analyzed, unless otherwise indicated. Missing data were not imputed. Adverse event analysis was done on the safety analysis set, defined as all participants in whom the subcutaneous EEG system was implanted.

Post-hoc analysis on the discomfort related to the implant was performed based on the per protocol set, defined as all participants in whom the subcutaneous EEG system was implanted and with no major protocol violations. Six participants answered the final question on discomfort from the implant after explantation and not before as explantation as intended. These answers were excluded.

## 3. Results

Baseline characteristics are shown in Table 1. For both the ULTS and SUBER trials, the genders were well balanced, and the participants spanned a wide age range. In the ULTS study all lead locations were over the temporal lobe, while the SUBER trial had implantations over temporal, fronto-temporal and central areas.

**Table 1:** Baseline characteristics of the participants implanted with the subcutaneous device.

	Number of participants		Age	Implant location					
	F	M	Years mean ± std [range]	L			R		
				Temporal	Fronto-temporal	Central	Temporal	Fronto-temporal	Central
ULTS	13	12	33 ± 12 [19–61]	14	0	0	11	0	0
SUBER	7	6	43 ± 13 [28–64]	1	7	1	1	3	0

### 3.1. The surgeon's assessment

**Figure 4:**
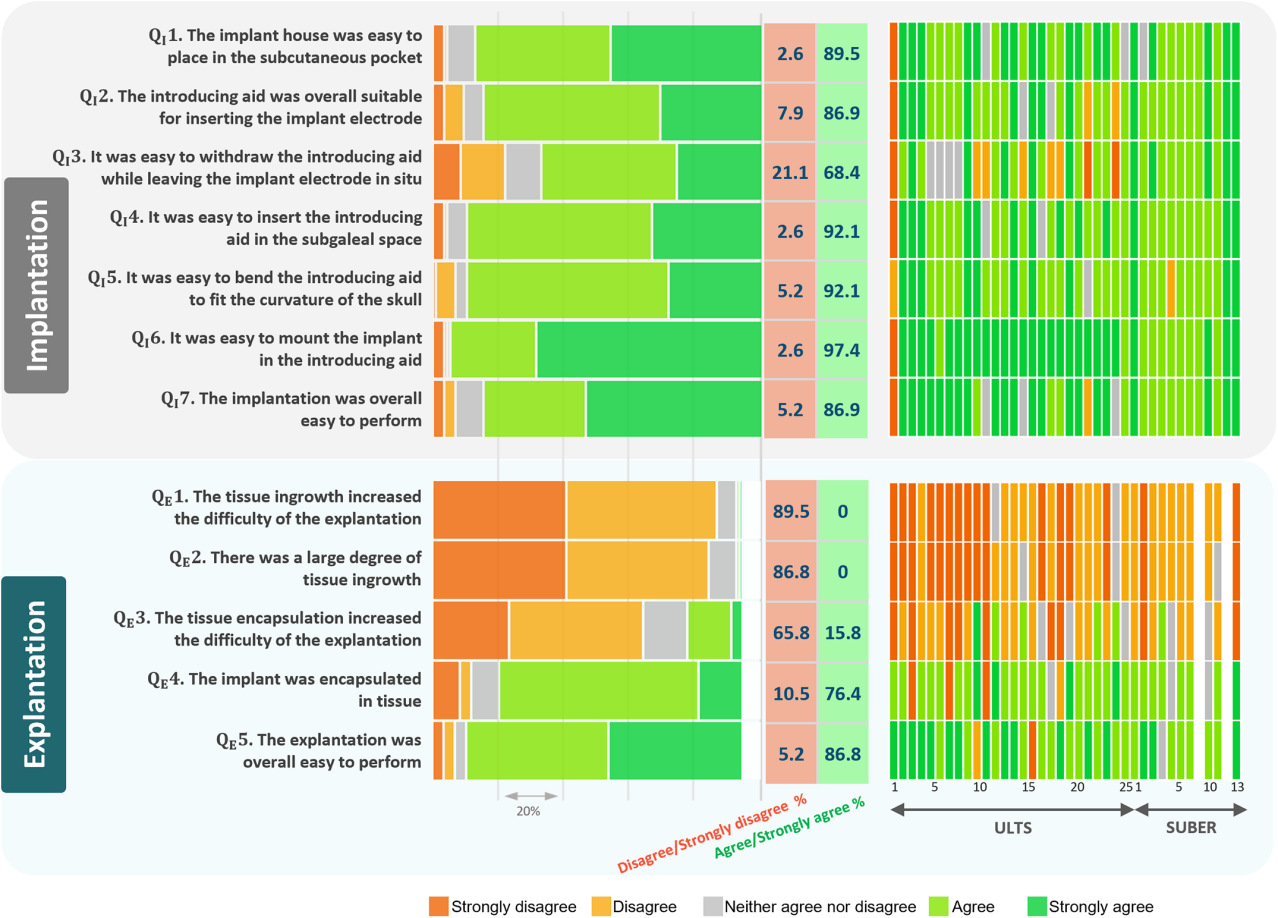
Surgeon satisfaction concerning implantation and explantation. Note that the first three questions to the explantation procedure (QE1–QE3) were asked so that strongly disagree or disagree was answered if surgeon was positive. Missing values are shown as white spaces.

With training, the implantation could be performed in approximately 15 min. Overall, the procedure was considered easy to perform (Figure 4) with only 2 (5.4%) cases of disagreements in QI7 (Figure 4). In these 2 cases (case 1 and 22 from ULTS), the implant was difficult to withdraw from the bended introducing needle and in one of the cases resulted in a reported adverse event because no extra implant was available.

It is apparent from Figure 4 that the general assessment was good, and no surgeons expressed concerns related to obtaining the desired position of the implant.

The explantation of the implant could be performed in a few minutes. Overall, the explantation procedure was considered easy to perform (Figure 4). However, in the ULTS study, the surgeon mentioned in the free text field of the questionnaire that for one explantation the silicone near the housing of the implant was damaged during the explantation and that the distal end of the electrode sticked to the tissue the last centimeter. Also, in the ULTS study it was mentioned in two cases that the device was encapsulated by soft tissue and in one case that the device was a little difficult to remove due to the capsule. In the SUBER study, it was explained in the free text field that the house was pulled but the silicone sheath of the lead was broken in one case. The lead was dissected, and the surgeon could pull it out without leaving any foreign bodies in the participant.

### 3.2. The participants' assessment

**Figure 5:**
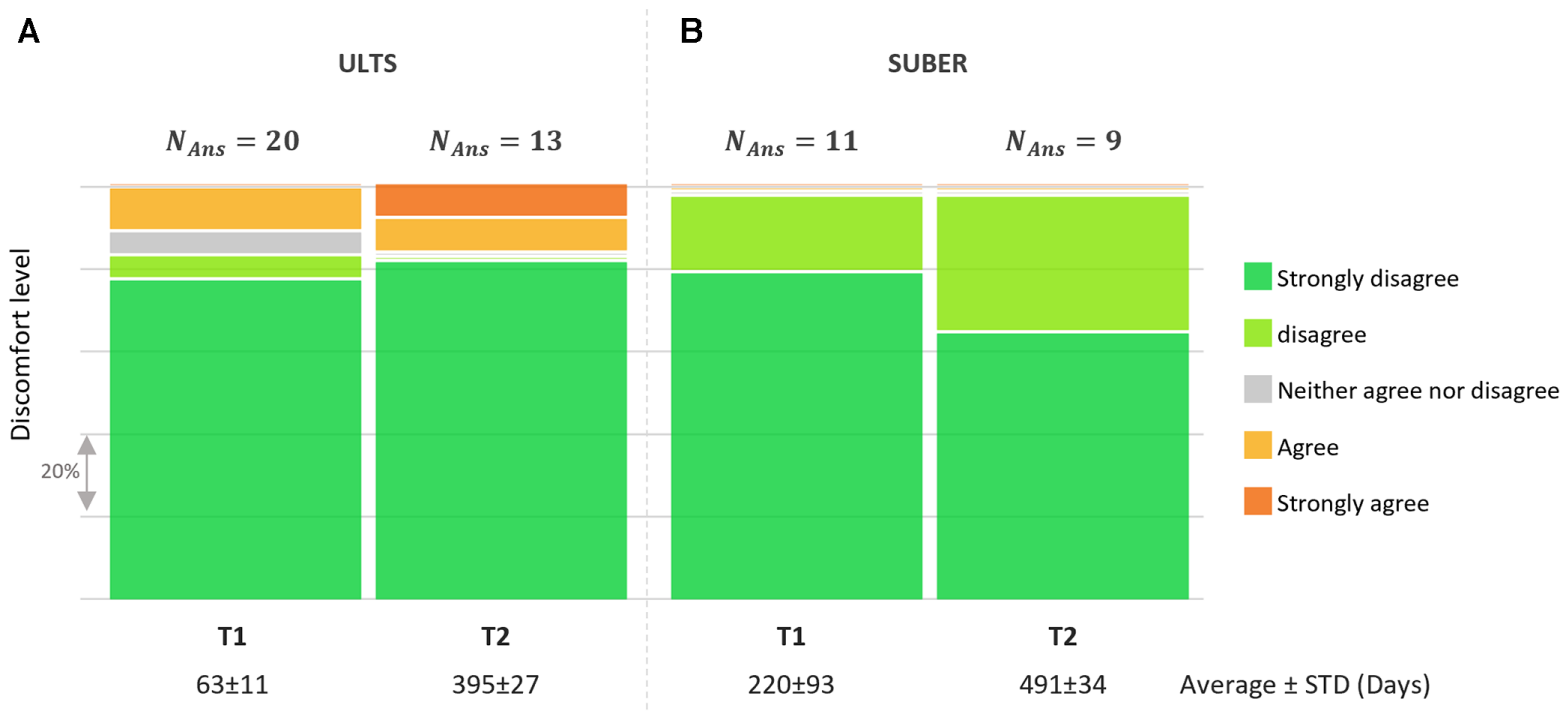
Answers to the question “I have experienced discomfort related to the UNEEG SubQ™ implant (e.g., pain, itching) for the last 2 days”. Only in the ULTS study did the participants report discomfort. NAns = the number of participant answers.

All participants were asked to respond to the statement: “I have experienced discomfort related to the UNEEG™ SubQ implant (e.g., pain, itching) for the last 2 days”. In the UTLS study, this was planned at 2 and 12 months after implantation, and in the SUBER study at 7 and 15 months after implantation (Figure 5). While 10% and 15% in the ULTS study reported discomfort related to the implant at 2 and 12 months, respectively, none of the participants in the SUBER cohort reported any discomfort. In free text, the discomfort was described as related to either itching, soreness, or tightness/irritation around the implant, sometimes causing headache. Those annoyances were all anticipated adverse device effects as stated in the instruction for use from the manufacturer.

### 3.3. Adverse events

A summary of adverse device effects related to the implantation according to the IMDRF definition are provided in Table 2. Note that each participant could contribute with the same adverse effect on several different occasions within the first 21 post-operative days.

**Table 2:** Adverse device effects related to the implantation reported from the ULTS and SUBER study. All AE reported up to 21 days after the implantation procedure were considered as having causal, possible, and/or probable relationship to the procedure or medical device. Each individual could contribute with the same adverse effect on several different occasions. Adverse device effects coding follows the IMDRF coding. *N* = number of participants, *n* = number of participants with an event.

Description	IMDRF definition	ULTS	SUBER	Total
Headache	Pain in various parts of the head, not confined to the area of distribution of any nerve	11	4	**15**
Implant pain	Pain localized to the site of the implanted device.	9	0	**9**
Post-Operative Wound Infection	Infection of a surgical skin incision.	3	0	**3**
Perforation	A hole or opening made through a membrane or other tissue or material.	0	1	**1**
Insufficient information	It is not clear whether any health effects occurred, or a health effect appears to have occurred but there is not yet enough information available to classify the clinical signs, symptoms and conditions.	1	0	**1**
No clinical signs, symptoms or condition	No patient involvement or, no observable clinical symptoms or a change in symptoms is identified in the patient.	1	0	**1**
**Total**		**25**	**5**	**30**
**Total number of implantations**		**25**	**13**	**40**
**Number of participants**		**18**	**4**	**22**

The majority of the adverse device effects were mild. In 6 out of 38 cases, adverse events contributed to the decision to explant and discontinue the study: 4 of these cases involved implant pain or headache; 1 case involved a post-operative local infection; and in 1 case the tip of the lead was placed too superficially, and skin perforation occurred a few weeks after implantation. This led to an unexpected explantation at the hospital, and therefore recorded as a serious adverse event. No further harm happened to the participant.

One perioperative adverse device effect was reported in the form of interrupted and postponed surgery. Here, the implant was fixed in the introducing needle when attempting to withdraw the needle and no extra implant was available at the department. For post-operative adverse events, the most frequent reported events were by far “Headache” and “Implant pain” as anticipated.

For the explantation procedure three adverse device effects were reported from three different participants. These were post-operative wound infection, headache and pain resulting in minor injury/illness/impairment. No serious adverse events were reported to be related to the explantation procedure.

## 4. Discussion

The current article represents the first presentation of the surgical and user related experiences in the use of the subcutaneous EEG system. Overall, we found the device to be safe and well tolerated in most individuals. Moreover, both the implantation and the explantation procedure were considered swift and easy by the surgeons.

According to the surgeon's assessment, the implantation procedure was generally considered easy to perform. However, in the ULTS study, bending the introducing needle in two cases hindered leaving the lead *in situ* when retracting the needle.

The explantation procedure was generally quick and easy. In some instances, we found tissue adherence to the silicone sheath close to the housing. Thus, special emphasis should be put on full separation between the implant and soft tissue, as forceful retraction might damage the silicone sheath of the lead.

In the questionnaires, only 2 participants in the ULTS study and none from the SUBER study reported discomfort. We suppose the primary reason for this group difference can be ascribed to the participants; The SUBER group consisted of people with epilepsy, who suffered from intractable seizures, and thus saw a direct potential benefit to gain control over their condition. On the other hand, the ULTS group consisted of healthy people without any experience of EEG recordings, nor of severe illness whose primary motivation was monetary compensation and potentially the reward of doing something good for science but presumably therefore had a lower tolerance towards a foreign body under the skin.

One perioperative adverse event was encountered. Here, the implantation procedure had to be interrupted and postponed getting a new implant adhered to as the first implant got stuck in the introducing needle. This event could have been avoided by keeping a spare implant at the site of implantation. Moreover, this was the first implantation to be performed in the ULTS study and the risk of this adverse events likely would have been reduced by a more thorough surgical training.

The most common post-operative adverse events were headache and pain located around the implant. Headache was reported at 11 instances in the ULTS study and 4 instances in the SUBER study. While pain located around the implant was registered 9 times in the ULTS study none of the individuals in the SUBER study reported pain although one reported continuous headache. The reason for the difference probably lies within a difference in the groups as described above. All instances apart from 4 leading to discontinuation, could be relieved with over-the-counter painkillers. The transitory pain/headache is anticipated as also stated in the instruction for use of the subcutaneous device, but we conclude that it is important to stress to the participant that some level of transient post-operative headache and pain around the implantation is to be expected.

Minimally invasive surgery for placement of the subcutaneous EEG implant, 24/7 EEG™ SubQ, is safe and the procedure is considered swift and easy to perform by both neuro- and ENT surgeons. The implant was well tolerated by most participants and without major adverse concerns. Minor instances of headache or pain around implant location is to be expected for up to 21 days post-surgery. Future developments on the device could advantageously be focused on optimizing the introducing needle.

## Scope statement

Subcutaneous EEG, which provides continuous brain wave recordings for months or years, has the potential to propel neurodiagnostic areas forward. Particularly epilepsy has received the initial attention from subcutaneous EEG investigations. While signal quality, automatic seizure detection performance and seizure forecasting are often subjects of investigation for the subcutaneous EEG devices, we have for the first time investigated the safety of the implantation and explantation of the subcutaneous system. We believe this subject will be of high interest for the readers of Frontiers in Neurology, as the expected benefits from the subcutaneous implant should always outweigh the potential disadvantages including the surgical procedure.

## Data Availability

The original contributions presented in the study are included in the article/Supplementary Material, further inquiries can be directed to the corresponding author.

## References

[1] Duun-Henriksen J Baud M Richardson MP Cook M Kouvas G Heasman JM A new era in electroencephalographic monitoring? Subscalp devices for ultra-long-term recordings. Epilepsia. (2020) 61:1805–17. 10.1111/epi.16630 32852091

[2] Haneef Z Yang K Sheth SA Aloor FZ Aazhang B Krishnan V Sub-scalp electroencephalography: a next-generation technique to study human neurophysiology. Clin Neurophysiol. (2022) 141:77–87. 10.1016/j.clinph.2022.07.003 35907381

[3] Pathmanathan J Kjaer TW Cole AJ Delanty N Surges R Duun-Henriksen J. Expert perspective: who may benefit most from the new ultra long-term subcutaneous EEG monitoring? Front Neurol. (2021) 12:817733. 10.3389/fneur.2021.817733 35126304 PMC8810530

[4] Gangstad SW Mikkelsen KB Kidmose P Tabar YR Weisdorf S Lauritzen MH Automatic sleep stage classification based on subcutaneous EEG in patients with epilepsy. Biomed Eng Online. (2019) 18:106. 10.1186/s12938-019-0725-3 31666082 PMC6822424

[5] Juhl CB Højlund K Elsborg R Poulsen MK Selmar PE Holst JJ Automated detection of hypoglycemia-induced EEG changes recorded by subcutaneous electrodes in subjects with type 1 diabetes–the brain as a biosensor. Diabetes Res Clin Pract. (2010) 88:22–8. 10.1016/j.diabres.2010.01.007 20074827

[6] Zaer H Deshmukh A Orlowski D Fan W Prouvot P-H Glud AN An intracortical implantable brain-computer interface for telemetric real-time recording and manipulation of neuronal circuits for closed-loop intervention. Front Hum Neurosci. (2021) 15:618626. 10.3389/fnhum.2021.618626 33613212 PMC7887289

[7] Lehnertz K Rings T Bröhl T. Time in brain: how biological rhythms impact on EEG signals and on EEG-derived brain networks. Front Netw Physiol. (2021) 1:755016. 10.3389/fnetp.2021.755016 36925573 PMC10013076

[8] Cook MJ O’Brien TJ Berkovic SF Murphy M Morokoff A Fabinyi G Prediction of seizure likelihood with a long-term, implanted seizure advisory system in patients with drug-resistant epilepsy: a first-in-man study. Lancet Neurol. (2013) 12:563–71. 10.1016/S1474-4422(13)70075-9 23642342

[9] Anderson WS Kossoff EH Bergey GK Jallo GI. Implantation of a responsive neurostimulator device in patients with refractory epilepsy. Neurosurg Focus. (2008) 25:E12. 10.3171/FOC/2008/25/9/E12 18759613

[10] Ryvlin P Rheims S Hirsch LJ Sokolov A Jehi L. Neuromodulation in epilepsy: state-of-the-art approved therapies. Lancet Neurol. (2021) 20:1038–47. 10.1016/S1474-4422(21)00300-8 34710360

[11] Brunnhuber F Amin D Nguyen Y Goyal S Richardson MP. Development, evaluation and implementation of video-EEG telemetry at home. Seizure. (2014) 23:338–43. 10.1016/j.seizure.2014.01.009 24512778

[12] Viana PF Remvig LS Duun-Henriksen J Glasstetter M Dümpelmann M Nurse ES Signal quality and power spectrum analysis of remote ultra long-term subcutaneous EEG. Epilepsia. (2021) 62:1820–8. 10.1111/epi.16969 34250608

[13] Duun-Henriksen J Kjaer TW Looney D Atkins MD Sørensen JA Rose M EEG signal quality of a subcutaneous recording system compared to standard surface electrodes. J Sens. (2015) 2015:e341208. 10.1155/2015/341208

[14] Weisdorf S Gangstad SW Duun-Henriksen J Mosholt KSS Kjær TW. High similarity between EEG from subcutaneous and proximate scalp electrodes in patients with temporal lobe epilepsy. J Neurophysiol. (2018) 120:1451–60. 10.1152/jn.00320.2018 29995605

[15] UNEEG^TM^ Subq User Manual Surgical Procedure. Rev. IFU-10001-7. Allerod: UNEEG^TM^ Medical (2020).

